# International consensus on clinical severity scale use in evaluating Niemann–Pick disease Type C in paediatric and adult patients: results from a Delphi Study

**DOI:** 10.1186/s13023-021-02115-6

**Published:** 2021-11-18

**Authors:** William Evans, Marc Patterson, Frances Platt, Christina Guldberg, Toni Mathieson, Jessica Pacey, the Core Working Group for the Delphi Study, Elizabeth Berry-Kravis, Nicole Farhat, Jordi Gascon, Tarek Geberhiwot, Paul Gissen, Roberto Giugliani, Caroline Hastings, Bénédicte Héron, Jackie Imrie, Simon Jones, Robin Lachmann, Eugen Mengel, Marc Patterson, Mercedes Pineda, Denny Porter, Heiko Runz, Miriam Stampfer, Michael Strupp, Mark Walterfang

**Affiliations:** 1 NPUK, Suite 2, Vermont House, Concord, Washington, Tyne and Wear, NE37 2SQ UK; 2 Primary Care Stratified Medicine (PRISM), Division of Primary Care, University of Nottingham, Nottingham, UK; 3 Departments of Neurology, Pediatrics and Medical Genetics, Mayo Clinic Children’s Center, Rochester, MN USA; 4 Department of Pharmacology, University of Oxford, Mansfield Road, Oxford, OX1 3QT UK; 5 Orphazyme A/S, Ole Maaløes Vej 3, 2200 Copenhagen N, Denmark; 6 67health, Sterling House, Fulbourne Road, Walthamstow, London, E17 4E UK

**Keywords:** Niemann–Pick disease Type C (NPC), Clinical Severity Scales, Delphi Study, Consensus Paper

## Abstract

**Background:**

Several scales have been developed in the past two decades to evaluate Niemann–Pick disease Type C (NPC) severity in clinical practice and trials. However, a lack of clarity concerning which scale to use in each setting is preventing the use of standardised assessments across the world, resulting in incomparable data sets and clinical trial outcome measures. This study aimed to establish agreed approaches for the use of NPC severity scales in clinical practice and research.

**Methods:**

A Delphi method of consensus development was used, comprising three survey rounds. In Round 1, participants were asked nine multiple-choice and open-ended questions to gather opinions on the six severity scales and domains. In Rounds 2 and 3, questions aimed to gain consensus on the opinions revealed in Round 1 using a typical Likert scale.

**Results:**

Nineteen experts, active in NPC paediatric and adult research and treatment, participated in this study. Of these, 16/19 completed Rounds 1 and 2 and 19/19 completed Round 3. Consensus (defined as ≥ 70% agreement or neutrality, given the study aim to identify the severity scales that the clinical community would accept for international consistency) was achieved for 66.7% of the multiple-choice questions in Round 2 and 83% of the multiple-choice questions in Round 3. Consensus was almost reached (68%) on the use of the 5-domain NPCCSS scale as the first choice in clinical practice. Consensus was reached (74%) for the 17-domain NPCCSS scale as the first choice in clinical trial settings, but the domains measured in the 5-domain scale should be prioritised as the primary endpoints. Experts called for educational and training materials on how to apply the NPCCSS (17- and 5-domains) for clinicians working in NPC.

**Conclusions:**

In achieving a consensus on the use of the 17-domain NPCCSS scale as the first choice for assessing clinical severity of NPC in clinical trial settings but prioritising the domains in the 5-domain NPCCSS scale for routine clinical practice, this study can help to inform future discussion around the use of the existing NPC clinical severity scales. For routine clinical practice, the study helps provide clarity on which scale is favoured by a significant proportion of a representative body of experts, in this case, the 5-domain NPCCSS scale.

## Introduction

Niemann–Pick disease Type C (NPC) is a devastating, rare neurodegenerative disease characterised by a defect that severely impedes cellular lipid trafficking [1]. Inherited in an autosomal recessive manner, individuals with NPC have mutations in one of two genes, *NPC1* or *NPC2*. Approximately 95% of affected individuals have mutations in *NPC1* [1]. As a result, cholesterol and sphingolipids accumulate within the endosomal/lysosomal system, degrading the central nervous system (CNS) and causing a diverse number of neurological symptoms depending on the patient’s age at onset. These symptoms may include cerebellar ataxia, dysarthria, dysphagia, cataplexy, seizures, dystonia, vertical gaze palsy, progressive dementia and death by 8–25 years of age [2].

The exact prevalence of NPC disease is difficult to calculate due to inadequate clinical awareness as well as the relative complexity of biochemical testing. However, it has been estimated to be 1 case per 100,000 live births [3]. The severe disabilities caused by NPC, particularly during the later stages of the disease, affect a patient’s entire family and optimal disease management requires highly specialised healthcare within a multidisciplinary care setting. Although NPC is not yet curable, knowledge on its pathogenesis has increased several-fold since the characterisation of the *NPC1* and *NPC2* genes. The focus of therapy remains symptom management, while advances are made in identifying effective disease-modifying treatments and investigational therapies.

The goal of the research into potential treatments for NPC is to develop drugs that are safe, effective and accessible to all members of the community. However, because NPC is an ultra-rare disease with considerable variability, designing and defining clinical trial inclusion criteria and endpoints can be challenging. Following a series of multidisciplinary discussions that culminated in an interactive workshop held at the Niemann Pick UK (NPUK) Annual Conference in 2019, with input from patients, clinicians, researchers, and industry representatives, it was agreed that there was a pressing need to develop a consensus on the use of existing NPC clinical severity scales in routine clinical practice and clinical trials. By determining such consensus, assessments across the world could be standardised to establish comparable data sets and demonstrate treatment efficacy through meaningful outcome measures.

Several scales have been developed and published over the past two decades but, essentially, all are based on a four-domain scale initially developed by Iturriaga et al. [4] (see Table 1). The present study aimed to establish consensus on the use of the clinical NPC severity scales listed in Table 1 in three different settings: routine clinical practice, clinical trial enrolment and clinical trial assessment. A Delphi method of consensus development was used to integrate anonymised perspectives from a group of international clinical experts with expertise in treating both paediatric and adult NPC patients and utilising scales to determine NPC severity. The Delphi method has proven to be a reliable measurement instrument to derive the opinion of a group of experts and evaluate the extent of agreement and to resolve any disagreement on a topic [5]. It has been widely used to establish a consensus across a range of subject areas. The study was coordinated as an iterative process of three surveys, with the questions in each round based on the previous round’s results.

**Table 1:** Six clinical NPC severity scales under investigation

Scale name	List of domains measured
17-domain NPC Clinical Severity Score (NPCCSS) [18]	The NPCCSS measures 17-domains: Nine major domains: ambulation, cognition, eye movement, fine motor, hearing, memory, seizures, speech, swallowing Eight minor domains: auditory brainstem response, behaviour, gelastic cataplexy, hyperreflexia, incontinence, narcolepsy, psychiatric, respiratory problems
5-domain NPCCSS [16]	Based on the 17-domain NPCCSS, the 5-domain NPCCSS measures ambulation, cognition, fine motor, speech and swallowing (five domains selected by NPC individuals, their caregivers and NPC experts as the most clinically relevant)
Disability Scale (NPC-specific) [4]	It measures four domains: ambulation, manipulation, language and swallowing, with scores 1–4 or 5
Disease-specific Disability Scale [19]	Adaption of the scale developed by Iturriaga et al. (2006) [4]. It measures four domains: ambulation, manipulation, language and swallowing, with weighted scores for each parameter on a scale from 0–1
NPC-cdb Scale [20]	Unlike previous scales, the NPC-cdb scale represents the sum of all past and current symptoms present in a patient at any given time, with each symptom contributing a severity-weighted summand
Functional Disability Scale [3]	Modified from Pineda et al. [19]. It measures seven domains: ambulation, manipulation, language, swallowing, eye movements, seizure and neurocognitive development (for patients under 12 years of age)

The objectives of this study were to build consensus among international experts in the field of NPC on: (i) the preferred clinical scale(s) for assessing NPC severity (ii) the most suitable NPC severity scale to be used within each of the following three settings: routine clinical practice, clinical trial enrolment and clinical trial assessment.

## Methods

### Study design

The Delphi technique is a reliable measurement instrument for developing novel concepts and setting the course of future-orientated research [6]. It assesses the opinion of a group of experts to gauge their levels of agreement and to resolve disagreement on an issue [5] and has been used successfully across a range of subject areas to gain a clinical consensus [7–9]. A Delphi study was carried out to gain a clinical consensus on six existing NPC clinical severity scales (see Table 1) that can be used within the following three settings: routine clinical practice, clinical trial inclusion criteria and clinical trial endpoints. A summary of the six severity scales and how they have been used in clinical practice and trials to date was shared with participants for their reference. Twenty experts were invited by email to participate and nineteen experts, active in NPC paediatric and adult research and treatment, participated in this study, all were known to be competent in English and all materials including the survey were conducted in English.

The Delphi technique is an iterative process that comprised three rounds. Participants were sent a link to an electronic survey for each round. Ahead of the first round of this Delphi study, participants received two documents: 1) Summary of the six existing clinical severity scales and 2) Clinical trials summary (see “Appendices”). Round 1 aimed to gather opinions on the use of the six severity scales and the key domains that should be measured in each clinical setting. Round 2 and 3 strived to gain consensus on these opinions. Ahead of Round 2, participants received the summary of the opinions revealed in Round 1. Anonymity was maintained for participants. Panel members were not made aware of the other panel members, except for MP a co-author and panel member, and participant identifiers were removed from the summary of opinions given to participants ahead of Round 2. This is an important consideration in Delphi studies to allow individuals to express their opinions freely and openly. However, the results of Round 2 were not shared ahead of Round 3 to avoid influencing the response.

#### Round 1

In Round 1, 16 specialists took part in a nine-question survey. Each of the nine questions constituted two parts: (a) a multiple-choice question and (b) a free-text question, that asked for reasoning, further insight or a recommendation based on their answer to part (a). The first round aimed to gather opinions on the six severity scales and domains that should be assessed in routine clinical practice, clinical trial inclusion criteria and clinical trial endpoints.

#### Round 2

In Round 2, 16 specialists, 11 of whom took part in Round 1, participated in an eleven-question survey. Participants were asked to independently rank nine statements using a 5-point Likert scale ('strongly agree', 'agree', 'neither agree nor disagree', 'disagree', 'strongly disagree'). The final two questions of the survey were free-text questions about the NPC severity scales. Consensus was determined as agreement, or neutrality, by greater than or equal to 70% of the participants.

#### Round 3

In Round 3, 19 experts took part in a six-question survey, which used the same 5-point Likert scale as in Round 2. The aim of this final round was to gain consensus on what should be recommended based on opinions from Rounds 1 and 2. Consensus was defined in the same way as in Round 2.

Three survey rounds are considered optimal when trying to reach consensus [10]. They also allow the free-text question responses in Rounds 1 and 2 to be incorporated into Rounds 2 and 3, respectively. All surveys were administered using SurveyMonkey and survey links were distributed via email.

### Consensus definition

Consensus was defined as greater than or equal to 70% of participants strongly agreeing/agreeing/neutrality on the Likert scale questions in Rounds 2 and 3. This level of agreement has been considered sufficient in several previous Delphi studies [11, 12]. Neutrality was included as a part of the consensus as the purpose was to identify the severity scales that the clinical community would accept for international consistency. Therefore, a neutral response implies that the individual would not be against the scale in question being adopted by the community and therefore willing to use.

### Core working group

The core working group was formed from key stakeholders who agreed to be involved at the NPUK annual conference in 2019. The group represents the patient community, TM, a parent of affected NPC children and an experienced international patient advocate and leader, and WE a parent of an affected child, with WE also having previous experience of conducting clinical surveys and consensus development; an internationally recognised NPC clinician, MP; an internationally recognised NPC researcher who co-developed an approach to NPC patient stratification, FP [16], a pharmaceutical industry expert in clinical outcomes CG, and a medical communications expert, JP.

### Survey development

The initial survey development involved the definition of a research question and development of the questions to be used in Round 1, based on the study team’s expertise and a review of the literature. This initial development was carried out by the Core Working Group. To meet the study objectives, the survey was split into three sections. The first round included questions to establish opinions on the most useful NPC severity scales and domains measured in each clinical setting and the second and third round aimed to gain consensus on the opinions gathered in Round 1.

### Expert panel recruitment

In Delphi studies, the minimum number of participants to be considered sufficient for achieving a consensus has been debated, with recent literature suggesting that larger sample sizes can deliver diminishing returns concerning the validity of the findings and that small panels of similarly trained experts in a specialist field provide stable results to support effective decision-making. [13–15] In a specialist rare disease area, such as NPC, reaching a prescribed minimum target poses a challenge due to the limited total potential pool of qualified participants. Nonetheless, 20 international specialists from Europe, the United States, Australia and South America were invited to complete the Delphi study, of which 19 agreed to participate. The professional community in NPC is very small, given the rarity of the disease, so the authors of the existing clinical severity scales that are still practising as NPC clinicians were also invited to take part. The participants were identified by Dr William Evans, Chair of NPUK, and ratified by the Core Working Group as key specialists in NPC around the world and invited via email to participate in this Delphi study. Dr Marc Patterson, as the only Core Working Group member who is also a practising NPC clinical specialist, also took part in the Delphi panel.

## Results

### Participants

Each survey round of this Delphi study comprised a representative panel of clinical experts (the Expert Panel) treating both paediatric and adult NPC patients, from seven different countries: United States of America (n = 6), United Kingdom (n = 5), Germany (n = 3), Spain (n = 2), Brazil (n = 1), France (n = 1) and Australia (n = 1). A little more than half (58%) of the study participants included in the study were paediatric specialists.

### Round 1

In Round 1, consensus was reached amongst the 16 international experts on the five most important domains to be measured to assess NPC clinical severity in the context of all three clinical settings (routine clinical practice, trial enrolment and clinical trial outcome measures). These included: ambulation, cognition, fine motor, speech and swallowing. Although these are the five domains captured in the 5-domain NPCCSS scale, the group was far from unanimous in the ambition to use a single scale across each of the clinical settings. Nonetheless, the 5-domain was among the highest-ranked for preferred use within all three settings: the top choice for 43.75% of participants for routine clinical use (versus 18.75% for the 17-domain NPCCSS, Disease specific disability scale and Functional disability scale); 37.5% for trial enrolment (second to the more granular 17-domain NPCCSS, chosen by 43.75 of participants); and 50% for clinical trial outcome measures (followed by the 17-domain NPCCSS preferred by 31.25% of participants). The most divisive question of the survey was regarding the adoption of a single severity scale in all scenarios, with some responses supportive of the consistency and optimisation of a scale on a global scale while others suggested that a single scale would be too reductive. Based on Round 1 results, detailed in Table 2, the second round focused on questions that asked participants to rate statements according to a typical Likert scale.

**Table 2:** Responses to statement included in Round 1 (16 respondents)

*1a. Which of the following NPC Severity Scales is the most useful as a practical measure of disease severity in normal clinical practice?*	*17-domain NPCCSS* [18]		*5-domain NPCCSS* [16]		*Disability scale* [4]			*Disease-specific disability scale* [19]		*NPC-cdb scale* [20]		*Functional disability scale* [3]			*None*		*Other*	
	18.75% (3)		43.75% (7)		12.5% (2)			18.75% (3)		0% (0)		18.75% (3)			0% (0)		6.25% (1)	
*1b. Please explain the reason for your answer*	**Summary of key insights:** The 5-domain NPCCSS and Disease-specific disability scale were highlighted by multiple respondents as being simple, quick to administer and complete in any clinical environment in a routine clinical exam, with no additional work, tools or expertise required The increased validity of the 17- and 5-domain NPCCSS scales, given their recent use by multiple groups for large cohorts of NPC patients and in clinical trials, was cited in further support of their use While the time-effectiveness and accuracy of the 5-domain NPCCSS scale in clinical practice were acknowledged, its limitations were also flagged in terms of evaluation of certain subsets of patients, e.g. those with mainly psychiatric involvement or experiencing seizures The granularity of scores and the comprehensiveness of the 17-domain NPCCSS scale was appreciated by multiple respondents Notably, the accuracy of the description of eye movement impairment was questioned across all of the scales The challenges of capturing progression in late-onset patients with more slowly-progressing disease when using these scales was also raised, with the suggestion for greater granularity of scoring across domains																	
*2a. In the context of routine clinical practice, if you had to limit measurements to only 5 of these domains, which would you select?*	*Ambulation*	*Cognition*		*Eye movement*		*Fine motor*			*Hearing*	*Memory*	*Seizures*			*Speech*		*Swallowing*		*Other*
	**100%** (16)	**100%** (16)		12.5% (2)		**93.75%** (15)			6.25% (1)	12.5% (2)	12.5% (2)			**81.25%** (13)		**87.5%** (14)		25% (4)
*2b. Please let us know which are the minimum number of domains you feel would be sufficient to reflect disease burden and progression in everyday practice and why*	*1–4 domains*						*5-domains*						*7–9 domains*					
	18.75% (3)						43.75% (7)						12.5% (2)					
*3a. Which of the following NPC Severity Scales is the most useful as a measure of disease severity for enrolment, in the context of a research study or clinical trial?*	*17-domain NPCCSS* [18]		*5-domain NPCCSS* [16]		*Disability scale* [4]			*Disease-specific disability scale* [19]		*NPC-cdb scale* [20]		*Functional disability scale* [3]			*None*		*Other*	
	43.75% (7)		37.5% (6)		0% (0)			12.5% (2)		12.5% (2)		6.25% (1)			6.25% (1)		6.25% (1)	
*3b. Please explain the reason for your answer*	**Summary of key insights:** The 17-domain NPCCSS scale was most popular among respondents in the context of clinical trial enrolment; it was seen as the most refined scale with the broadest coverage of the disease and the largest score range in each domain (5 instead of 4 or less). However, it was noted that the scale could be improved with respect to the linearity of the rating in some domains Granularity was seen as critical to measuring change and baseline assessment within clinical trials; it should be as comprehensive as possible while remaining quantifiable As more data becomes available, e.g., genomic data, there may be a need to reconsider which parameters are most important and whether preferred scales need to be amended accordingly Simplicity was seen as valuable for multi-center trials. The simplicity of the 5-domain NPCCSS scale, as well as its proven correlation with the 17-domain scale, may drive the preference for use in some trials Additionally, the question of which parameters can be expected to change in a clinical trial should be considered, as they determine both the endpoint and inclusion criteria and the identification of patients who can demonstrate measurable progression. Given the heterogeneity of the condition, general scores may not be suitable for every trial																	
*4a. In the context of trial enrolment, if you had to limit measurements to only 5 of these domains, which would you select?*	*Ambulation*	*Cognition*		*Eye movement*		*Fine motor*			*Hearing*	*Memory*	*Seizures*			*Speech*		*Swallowing*		*Other*
	**100% (16)**	**93.75% (15)**		25% (4)		**93.25% (15)**			0% (0)	18.75% (3)	18.75% (3)			**87.5% (14)**		**87.5% (14)**		6.25% (1)
*4b. Please let us know which are the minimum number of domains you feel would be sufficient to reflect disease burden and progression in a clinical trial setting and why*	*1–4 domains*						*5-domains*						*7–9 domains*					
	12.5% (2)						43.75% (7)						12.5% (2)					
*5a. Do you think that the ASIS score (Annual Severity Incremental Score), is a suitable measure to capture the rate of disease progression for trial enrolment and/or clinical trial outcome measures?*	*Yes, it is suitable for trial enrolment*						*Yes, it is suitable for clinical trial outcome measures*						*No, it is not suitable for either*					
	68.75% (11)						62.5% (10)						12.5% (2)					
*5b. Please explain the reason for your answer*	**Summary of key insights:** Multiple respondents highlighted that as ASIS is a general scale and should only be a secondary outcome measure. It is not as sensitive as other scales, particularly over a potentially short period of a clinical trial Broadly, its value for both prospective and retrospective measures was recognised by the majority of respondents, particularly in regard to quantifying progression in a respective age group over multiple years of treatment The need for more data on its use was highlighted by two respondents It was seen by two separate respondents as a better indicator of disease progression than age of onset and arguably the best scale available for this																	
*6a. Do you think an NPC severity score is a suitable endpoint for a clinical trial? If 'yes', which of the following NPC severity score systems is optimal?*	*17-domain NPCCSS* [18]		*5-domain NPCCSS* [16]		*Disability scale* [4]			*Disease-specific disability scale* [19]		*NPC-cdb scale* [20]		*Functional disability scale* [3]			*None*		*Other*	
	31.25% (5)		50% (8)		0% (0)			6.25% (1)		6.25% (1)		12.5% (2)			0% (0)		25% (4)	
*6b. Please explain the reason for your answer*	**Summary of key insights:** To market an expensive drug, a sponsor will need to demonstrate a positive impact on the dynamics of a composite clinical progression score The challenge of conducting an outcome trial of sufficient duration (probably > 24mo) to see a robust statistically significant clinical effect in any of the scales with a reasonable number of participants was raised by more than one respondent A severity score was seen as a suitable outcome measure if the data are collected properly and in a rigorous and consistent manner across sites and with proper (and fairly simple) training. Otherwise, data are less reliable and more objective measures are needed, such as MRI, BAEPS, oxysterols, and videos with blind raters, of walking and the 9HPT, as suggested by other respondents To support reproducibility and reliability across trial sites, limiting the severity score to the 5 major domains was seen as sensible. These need to be guided with precise assessments (named tests) and be age/cognition dependent The 5-domain scale addresses the five most important domains, based on clinician and family opinion, and does not include items that can vary due to other treatments and thus act as confounders																	
*7a. Which do you think are the key domains to capture as a clinical trial outcome measure? Please select all that apply*	*Ambulation*	*Cognition*		*Eye movement*		*Fine motor*			*Hearing*	*Memory*	*Seizures*			*Speech*		*Swallowing*		*Other*
	**93.75% (15)**	**75% (12)**		25% (4)		**100% (16)**			12.5% (2)	25% (4)	25% (4)			**87.5% (14)**		**81.25% (13)**		12.5% (2)
*7b. Please provide any further insights*	**Summary of key insights:** The top 5-domains chosen by the group were seen as the most relevant to describe neurological disease progression. However, it was suggested the impact of seizures needs to be accounted for, as well as the quality of life of the patient and their caregivers Sophisticated computer assessment to measure speech in trials was suggested for consideration Until an effective disease modifying therapy becomes available, deciding what to measure in clinical trials remains a challenge. The solution proposed was to start by measuring everything and adapting endpoints dependent on the findings, particularly with different age groups involved																	
*8a. Do you think that the adoption of a single severity scale in all scenarios is optimal, even if this means losing some refinement? If 'yes', which of the following NPC Severity Scales would you recommend?*	*17-domain NPCCSS* [18]		*5-domain NPCCSS* [16]		*Disability scale* [4]			*Disease-specific disability scale* [19]		*NPC-cdb scale* [20]		*Functional disability scale* [3]			*None*		*Other*	
	12.5% (2)		50% (8)		0% (0)			6.25% (1)		0% (0)		12.5% (2)			25% (4)		0% (0)	
*8b. Please provide any further insights*	**Summary of key insights:** This was the most divisive question for the group, with many calling for greater consistency and optimisation of a single multi-domain scale on a global scale, while others suggested the use of a single scale would be too reductive. The following (sometimes conflicting) considerations were put forward: In the absence of a proven composite score that can work in all settings, the use of different scales in clinical trials should be at the liberty of each investigator/sponsor Neither clinical research nor clinical practice should be compromised by a one size fits all approach. This would be regression to the least common denominator Losing refinement of scales may be acceptable in some clinical routine practices but not in a trial setting. Even though an extensive set would be optimal the practicability may be less likely Alternatively, it may be appropriate to consider that if a scale cannot be implemented in routine clinical practice, it is not justifiable to use in a trial It is critically important to try to standardize scoring and implementation to make datasets comparable The 5-domain NPCCSS scale would be best suited to all three settings																	
*9a. Are a limited number of domains sufficient to meet needs in all scenarios? If 'yes', tick the limited number of domains you believe would be sufficient*	*Ambulation*	*Cognition*		*Eye movement*		*Fine motor*			*Hearing*	*Memory*	*Seizures*			*Speech*		*Swallowing*		*Other*
	50% (8)	43.75% (7)		12.5% (2)		50% (8)			0% (0)	0% (0)	6.25% (1)			37.5% (6)		43.75% (7)		6.25% (1)
*9b. Please provide any further insights*	**Summary of key insights:** Many respondents did not agree with the question that a limited number of domains could be sufficient to meet the needs all scenarios It was suggested that there should be a focus on domains where change can be expected with therapy and a domain where changes can be quantified. Measuring everything at baseline within clinical trials would show where changes occur The five identified domains are almost always all involved as the disease progresses. Only a small percentage of patients experience hearing loss and seizures, memory is a part of general cognition and too hard to separate from this domain, and eye movement change are difficult to measure In very young children, an additional developmental scale (e.g. Bayleys, Kauffman, etc.) should be used and, in adults, a dementia scale should be used One respondent suggested that it remains unclear if breaking down scores into domains is particularly helpful, while the dynamics of additive sum scores (across domains or without domains) is what matters for outcome trials An additional suggestion included videoing of the walk-test and 9HPT as functionally most relevant; with analysis performed by blinded raters on a 0 + / − 3 scale																	

Numbers highlighted in bold indicate questions/statements for which consensus was achieved (greater than or equal to 70% agreement of neutrality)

### Round 2

In Round 2 consensus was achieved amongst 16 of the experts for six of the nine statements (see Table 3). The panel of experts agreed that it was ‘desirable’ (81%) and ‘achievable’ (75%) to determine a single, standardised NPC clinical severity scale for routine clinical practice and clinical research on a global scale within the scope of the existing scales. Further, 100% of respondents agreed that a clinical paper recommending which NPC clinical severity scale should be used in each clinical setting would be valuable to the international clinical and patient community. Consensus was also reached on the statement that the domains measured in the 5-domain scale provided an accurate clinical understanding of NPC severity in clinical practice and trials (87%) and, if there was only one international scale recommended for use evaluating the disease, it would be the 5-domain NPCCSS (81%).

**Table 3:** Responses to statement included in Round 2 (16 respondents)

Question	Round 2	
	*Agree/neutral*	*Disagree*
*1. A single, standardised NPC clinical severity scale that can be used in routine clinical practice as well as clinical research on a global scale is desirable*	**81% (13)**	19% (3)
*2. A single, standardised NPC clinical severity scale that can be used in routine clinical practice as well as clinical research on a global scale is achievable within the scope of existing scales*	**75% (10)**	25% (6)
*3. A clinical consensus paper recommending which NPC clinical severity scale to use per different clinical setting (comprising routine practice and trial research) would be valuable to the international clinical and patient community*	**100% (16)**	0% (0)
*4. Assessment across the following 5-domains, provides an accurate clinical understanding of NPC severity: Ambulation, Cognition, Fine motor, Speech, Swallowing*	**87% (14)**	13% (2)
*5. If only one existing NPC severity scale was to be used for the evaluation of disease in normal clinical practice internationally, I would recommend the 5-domain NPCCSS scale*	**81% (13)**	19% (3)
*6. It is essential to measure all 17-domains in the NPCCSS during a clinical trial to capture all potential treatment benefits for people living with NPC*	69% (9)	31% (7)
*7. It is sufficient to measure the 5-domains in the 2018 NPCCSS during a clinical trial to capture relevant potential treatment benefits for people living with NPC*	**75% (10)**	25% (6)
*8. I believe the 5-domain NPCCSS scale satisfies requirements for use in all clinical settings, to standardise assessments on a global scale*	69% (9)	31% (3)
*9. I believe it is feasible and there is a need to develop a new NPC clinical severity scale that satisfies requirements for use in all clinical settings, to standardise assessments on a global scale*	31% (7)	69% (9)
*10. If a new universal NPC clinical severity scale were to be developed, the most important way that it would differ from existing scales would be…*	**Summary of key insights:** To balance breadth with brevity and usability To focus on domains where change can be expected with disease progression or therapy To evaluate cognition at different ages To include quality of life measures To determine the impact of epilepsy To incorporate video of the performance of patients during the 9HPT and 8-min walk test To include age/subtypes-dependant items (e.g. epilepsy and cataplexy in late infantile-juvenile, psychiatry in adolescent-adult…) Based on the largest possible source data from natural history cohorts as well as clinical trials and take into account that NPC manifests and progresses differently across age groups and patient populations Used across regions, languages and cultures	
*11. What would be your recommendations to implement a more uniform approach to the use of NPC clinical severity scales?*	**Summary of key insights:** To publish a systematic review of the current scales and consensus To publish an expert consensus on which scale is preferred for clinical routine practice and which for trials To develop detailed SOPs and training on the use of severity scales To select a simple scale that can be used in different setting and is sensitive enough to capture the impact of the disease in the NPC patient To add QoL measures to 5-domain NPCCSS To gain insights from the community on what matters to patients and carers To provide patients with score sheets, a booklet or app, to complete regularly and which they present to their doctors at every appointment To include clinical scale biochemical markers and neuroimaging To evolve clinical scales with available data and distinct uses (e.g. in a specific NPC sub-population, or to track changes in a specific subject), particularly as personalised medicine is a goal of this decade To capture real-world results of scales systematically (e.g. INPDR) so that pre/post treatment effect are comparable	

Numbers highlighted in bold indicate questions/statements for which consensus was achieved (greater than or equal to 70% agreement of neutrality)

Two further statements narrowly missed reaching a consensus by 1% (69% consensus respectively). These related to whether it was essential to measure all 17-domains during a clinical trial and whether the 5-domain scale satisfies the requirements for use in all clinical settings. The final statement on which consensus was not reached related to the feasibility and need to develop a novel NPC clinical severity scale that satisfies requirements for use in all clinical settings.

The key themes of the responses about a new, universal NPC clinical severity scale (Question 10) included: a need to incorporate quality of life measures, age/subtype dependant items (such as epilepsy and cataplexy in late infantile-juvenile) and a video of patient performance during a 9-Hole Peg Test (9HPT) and 8-min walk test. When asked for recommendations to implement a more uniform approach to the use of NPC severity scales, participants suggested a published systematic review of the current scales, a published expert consensus, the inclusion of biochemical markers and neuroimaging, and to provide more agency to each patient (such as an app to fill in regularly) to help the doctors achieve personalised treatment. The key insights from the open-ended questions in Round 2 are summarised in Table 3.

### Round 3

In Round 3, consensus was reached on five out of the six statements (see Table 4). Despite consensus (81%) achieved during Round 2 that the 5-domain NPCCSS scale was the preferred scale for routine clinical practice and trials, the suggested recommendation in Round 3 that this be positioned as the first-choice scale in routine clinical practice, did not quite reach consensus (68%). However, the panel of 19 experts agreed that the 17-domain NPCCSS scale should be recommended as the first choice to assess the severity of NPC in clinical trial settings, with the domains listed in the 5-domain scale prioritised as the primary endpoints (74%). Furthermore, 74% of respondents agreed that there is no need for a new universal scale for all settings to be developed. However, resources or training on how to apply the NPCCSS (17- and 5-domains) should be developed and provided to clinicians working in NPC (89%). Further, 84% agreed that the consensus paper should be reviewed every five years to ensure that recommendations remain accurate.

**Table 4:** Responses to statement included in Round 3 (19 respondents)

Question	Round 3	
	*Agree/neutral*	*Disagree*
*1. The 5-domain NPCCSS scale is the first choice for assessing clinical severity of NPC in routine clinical practice*	68% (13)	32% (6)
*2. The 17-domain NPCCSS scale is the first choice for assessing clinical severity of NPC in clinical trial settings, prioritising the domains in the 5-domain scale (e.g. as primary endpoints)*	**74% (14)**	26% (5)
*3. There is no need for a new universal scale for all settings to be developed*	**74% (14)**	26% (5)
*4. Resources/training on how to apply the NPCCSS (17- and 5-domains) should be developed and provided to clinicians working in NPC*	**89% (17)**	11% (2)
*5. The consensus paper is reviewed periodically to ensure that its recommendations remain accurate*	**100% (19)**	0% (0)
*6. The timescale for periodic review of the consensus paper should be every 5 years*	**84% (16)**	16% (3)

Numbers highlighted in bold indicate questions/statements for which consensus was achieved (greater than or equal to 70% agreement of neutrality)

## Discussion

This Delphi study achieved consensus during Round 2 that the domains measured in the 5-domain NPCCSS scale provided an accurate clinical understanding of NPC severity. If there was only one international scale recommended for use in routine clinical practice, the respondents would recommend use of the 5-domain NPCCSS scale. Although this statement achieved consensus in Round 2, amongst a panel of 16 NPC specialists who completed the first two rounds, it did not quite reach consensus in Round 3 from a panel of 19 experts.

In Round 1, respondents highlighted the 5-domain NPCCSS scale as simple, accurate and quick to administer and complete in a routine clinical examination and that its simplicity was valuable for multi-centre trials to support reproducibility and reliability across sites. Further, it was noted that the domains measured in the 5-domain scale are present in nearly all cases of NPC as the disease develops, unlike hearing loss and seizures, which are typically present in only a small percentage of patients. Respondents also noted that the domains measured in the 17-domain scale posed several challenges. For example, as a domain, memory is difficult to separate from the cognition domain and that measuring changes in the eye movement domain can be problematic.

However, the 5-domain scale was seen as insufficient for evaluation of specific subsets of patients, such as those with mainly psychiatric involvement or experiencing seizures. Moreover, answers in Round 1 stressed the importance of the granularity of scores and the comprehensiveness provided by the 17-domain NPCCSS scale, in capturing the progression of late-onset patients with a slowly progressing disease, as well as for measuring change and baseline assessment in clinical trials. This likely led to the 74% consensus in Question 2 of Round 3 that the 17-domain NPCCSS should be the first-choice severity scale in clinical trial settings.

Given these insights, the Core Working Group recommends that the 17-domain NPCCSS is used as the preferred scale to assess NPC severity across clinical trial enrolment and trial outcome measures. However, the domains listed in the 5-domain scale (ambulation, cognition, fine motor, speech and swallowing) should take precedence as primary endpoints as they are the most relevant to describe neurological disease progression and quality of life [16]. As supported by the experts in Round 1, use of the 5-domain NPCCSS is recommended in multi-centre trials to support reproducibility and reliability of results across multiple trial sites. Lastly, the Core Working Group recommends that the 5-domain NPCCSS scale is used within routine clinical practice to assess the clinical severity of NPC patients. These recommendations provide greater global consistency and optimisation of both the 17- and 5-domain NPCCSS scales, whilst not becoming too reductive, which was noted as important by respondents in Round 1.

The Core Working Group also recommends that resources or training on the NPCCSS scales (17- and 5-domains) should be developed and provided to clinicians working with NPC patients to optimise the standardisation of their application. Further, it is advised that this consensus paper should be reviewed every five years to ensure that the recommendations remain accurate.

This Delphi study gathered consensus on the use of six existing NPC clinical severity scales, the findings for which have enabled the research team to deduce several significant recommendations and areas for further development. Drawing on an international panel of NPC clinicians, who treat both paediatric and adult NPC patients, views were gathered from a select, yet representative panel of experienced experts in the field. However, the rarity of NPC disease means that there is a limited global community of NPC specialists. As a result, the size and composition of the expert panel may reduce the generalisability of the results, and consideration should be given in future international consensus work to ensure the panel’s composition represents the global NPC community with if necessary, the inclusion of translated materials into the participants first language to reduce potential bias. Nonetheless, the final sample size (16 participants in Round 1 and 2 and 19 participants in Round 3) was greater than broadly accepted sufficient panel size of 10–15[17]. Given the global scale upon which this field operates, the Delphi consensus method, which can be conducted quickly and online, was an appropriate tool for collecting responses. In addition to identifying the areas of consensus, the study highlighted areas where there is less certainty in the field, such as balancing the need for greater consistency of a single, global multi-domain scale with the concern of becoming too reductive.

While a strength of the study was its ability to access an international network of specialists in the field of NPC research and treatment, some of the participants included in the study were those who developed the clinical severity scales under evaluation. The strong opinions from these participants may therefore have introduced some response bias. Further, it is acknowledged that the concept of ‘consensus’ is fairly fluid. While we have consensus, there are still experts among the group who strongly disagree with the recommendations and hold these views firmly. Given the small size of the expert community, research is unlikely to ever to reach consensus across all statements. However, the fact that 19 out of 20 invited participants took part in the Delphi study highlights both the perceived importance of this piece of work to the NPC community, and the influential role that patient groups can have in bringing together stakeholders for such projects. According to guidance from the National Institute for Health Research (NIHR) Health Technology, the Delphi technique typically results in a 20% dropout rate over the three rounds of consensus development. In this study, there was an absence of dropouts in any of the three rounds, therefore substantiating the validity of our recommendations.

A key limitation of this study is that it does not offer definitive guidance, as consensus in Round 2 on the 5-domain NPCCSS as the preferred scale for routine clinical practice did not reach final consensus in Round 3. This may be a result of nuances in question phrasing, or the use of a 5-point Likert scale, the use of a 9- or 10-point scale in future studies may provide a more sensitive measure to draw more nuanced conclusions.

However, the insights obtained were adequate to make several reliable recommendations. As a result, this consensus might facilitate a platform to enable standardisation of data capture and agreement on use for outcome measures.

We believe this study can help to inform and position future discussion around the use of the existing NPC clinical severity scales in clinical practice and trials. As more data, including genomic data, for NPC become available, the findings will become even more important and there may be a need to reconsider which parameters are most important and whether the preferred scales should be amended accordingly. Similarly, outcomes of ongoing trials of disease-modifying therapies for NPC will drive the need to identify the most appropriate clinical severity scale for determining drug efficacy.

## Conclusion

Within this Delphi study, experts confirmed that there was no need for a new universal scale for all settings to be developed. However, they highlighted a need to strike a balance between greater optimisation of a global, single multi-domain scale and it becoming too reductive when choosing between the six existing scales. Although consensus was achieved in Round 2 on the 5-domain NPCCSS as the preferred scale for routine clinical practice, this did not achieve a final consensus in Round 3. Given the small size of the expert community, research is unlikely to ever reach consensus across all statements. However, several meaningful recommendations could be drawn from the study. In line with the consensus achieved in Round 3, this study recommends the use of the 17-domain NPCCSS scale across clinical trial settings, but the five domains measured in the 5-domain scale should be prioritised as primary endpoints. Further, this study recommends the use of the 5-domain NPCCSS scale in routine clinical practice. The findings also indicate a need to develop educational and training materials on how to apply the NPCCSS (17- and 5-domains) for clinicians working in NPC.

## Data Availability

The datasets used and/or analysed during the current study are available from the corresponding author on reasonable request.

## Appendix 1: Summary of existing clinical severity scales

The following table provides a summary of the recognised scales used to evaluate the severity of Niemann–Pick disease Type C (NPC). A brief overview of the domains measured by each scale is provided, as well as insights as to how the scales have been used to date, including their use in ongoing clinical trials.

**Table:**

Scale name	List of domains measured	How the scales have been used to date				
		Use in clinical practice	Patient registries	Recruitment of patients into clinical trials	Outcomes measures in clinical trials	Additional notes
Disability Scale (NPC-specific) [4]	The Disability Scale was developed via a cohort of 30 NPC patients It measures four domains: ambulation, manipulation, language and swallowing, with scores 1–4 or 5	No information available	No information available	No information available	No information available regarding use in clinical trials to date	Used in a study that examined the structure of the callosum in a group of adult patients with NPC and compared callosal structure with a group of matched controls, and to relate callosal structure with state and trait illness variables [21]
Disease-specific Disability Scale [19]	In an adaption of the scale developed by Iturriaga et al. (2006) [3], the Disease-specific Disability Scale assigns weighted scores for each parameter on a scale from 0–1 It measures four domains: ambulation, manipulation, language and swallowing	This study incorporates findings from an observational retrospective cohort study conducted to further assess the effects of miglustat on neurological disease progression in NPC patients treated with miglustat in the clinical practice setting, outside the context of clinical trials	The authors anticipated that this scale would be included as one of the standard monitoring assessments in the planned international disease registry for NPC patients and yield further, valuable long-term information on the utility of the scale in monitoring disease progression and treatment response	No information available	Primary outcomes in the study: *Clinical experience with miglustat therapy in pediatric patients with Niemann–Pick disease type C: a case series* [22] modified with scores to calculate an overall (composite) disability score	Used to evaluate the efficacy and course of disease in patients treated with miglustat using two neuroimaging modalities [23] Used in a study to identify retinal degeneration in NPC1-disease and to investigate possible subclinical retinal degeneration in NPC1-MC [24]
NPC Clinical Severity Score (NPCCSS) [18]	Comprises 17-domains based on a cohort of 18 then-current NPC patients and 19 historical cases from the National Institutes of Health The NPCCSS measures: nine major domains: ambulation, cognition, eye movement, fine motor, hearing, memory, seizures, speech, swallowing eight minor domains: auditory brainstem response, behaviour, gelastic cataplexy, hyperreflexia, incontinence, narcolepsy, psychiatric, respiratory problems	No information available	No information available	According to the authors, the ability to combine data from patients of variable age of onset will facilitate recruitment for clinical trials	Primary outcomes in the study: *Long-Term Treatment of Niemann–Pick Type C1 Disease With Intrathecal 2-Hydroxypropyl-β-Cyclodextrin* [25] Secondary outcomes in the study: *Intrathecal 2-hydroxypropyl-β-cyclodextrin decreases neurological disease progression in Niemann–Pick disease, type C1: a non-randomised, open-label, phase 1–2 trial* [26] Primary outcomes in the study: *VTS-270 for the treatment of Niemann–Pick disease type C. Molecular Genetics and Metabolism* [27] Primary outcomes in the study: *Arimoclomol Prospective Study in Patients Diagnosed With Niemann Pick Disease Type C* [28] Primary objectives in the study: *Clinical disease progression and biomarkers in Niemann–Pick disease type C: a prospective cohort study* [29]	Evaluated whether the lower corpus callosum fractional anisotropy, volume, and cross-sectional area significantly correlate with higher severity score in patients with NPC [30] Used to systematically describe the neurocognitive phenotype of children and adolescents with NPC1, identifying heterogeneity and decline, aiding in understanding the natural history of the disease to plan treatment studies [31]
NPC-cdb Scale [20]	Unlike previous scales, the NPC-cdb scale represents the sum of all past and current symptoms present in a patient at any given time, with each symptom contributing a severity-weighted summand	The authors note that the scale’s ease of use should prove useful in clinical settings. It could also complement the widely used, but less comprehensive, scales that only poorly reflect the heterogeneous clinical picture of NPC	This is used in the INPDR registry for registering NPC symptoms at baseline and how they evolve over time	No information available	Primary outcomes in the study: *Arimoclomol Prospective Study in Patients Diagnosed With Niemann Pick Disease Type C* [28] Primary objectives in the study: *Clinical disease progression and biomarkers in Niemann–Pick disease type C: a prospective cohort study* [29]	
ASIS [16]	The Annual Severity Increment Score (ASIS) measures rate of disease progression using Yanjanin et al.’s (2009) scale [18] The only data required to calculate ASIS is the total severity score and the precise age of the patient when the score was ascertained	Authors denoted that their annual severity increment score (ASIS), that measures rate of disease progression, could easily be used in clinical practice	Anticipated contribution to pre-trial longitudinal data for individual patients held by patient registries (International Niemann–Pick Disease Alliance)	Authors note that ASIS provides an evidence-based stratification/ recruitment tool that is easy to calculate and apply in any clinical setting	Secondary outcomes in the study: *Application of N-palmitoyl-O-phosphocholineserine for diagnosis and assessment of response to treatment in Niemann–Pick type C disease* [32]	Validated in an observational clinical study in NPC patients treated with the drug Tanganil (acetyl-DL-leucine)
Severity rating scale of neurological manifestations and dysphagia [33	A clinical scoring scale for a series of neurological parameters. Developed to measure the treatment efficacy of miglustat It measures six domains: gait abnormalities, dysmetria, dystonia, dysarthria developmental delay/cognitive impairment and dysphagia	No information available	No information available	No information available	Primary outcomes in the study: *Long term follow-up to evaluate the efficacy of miglustat treatment in Italian patients with Niemann–Pick disease type C* [33]	
5-Domain NPCCSS [16]	Based on the 17-domain NPCCSS, the 5-domain NPCCSS measures ambulation, cognition, fine motor, speech and swallowing Five domains, selected by NPC individuals, their caregivers and NPC experts as the most clinically relevant, reduce variability and increase the suitability for use in clinical trials	The authors note that when combined, these five domains correlated well with total severity, suggesting they may be the most relevant domains to analyse in clinical trials with direct QoL relevance	No information available	No information available	Primary outcomes in the study: *Arimoclomol Prospective Study in Patients Diagnosed With Niemann Pick Disease Type C* [28] Primary objectives in the study: *Clinical disease progression and biomarkers in Niemann–Pick disease type C: a prospective cohort study* [29]	
Functional Disability Scale [3]	Modified from Pineda et al. (2009) [19], this clinical severity assessment measures seven domains: ambulation, manipulation, language, swallowing, eye movements, seizure and neurocognitive development (for patients under 12 years of age) However, it has not been formally validated for treatment monitoring	The authors note that these guidelines can inform care providers, care funders, patients and their carers of best practice of care for patients with NPC	Backed by expert physicians, geneticists, allied healthcare professionals and patient support groups involved in the International Niemann–Pick Disease Registry (INPDR) project (www.inpdr.org), which is supported by the EU Directorate General for Health and Consumers (DG-SANCO) via the Consumers, Health, Agriculture and Food Executive Agency (CHAFEA)	No information available	No information available regarding use in clinical trials to date	

## Appendix 2: Clinical trials summary

The following table provides a summary of NPC clinical trials and the scales used for primary and secondary outcomes measures at a glance.

**Table:**

Trial name	Primary outcome measure	Secondary outcome measure
Application of Miglustat in Patients With Niemann-Pick Type C [34]	Functional Disability Scale [3]—It was used if VFSS outcome measure could not be performed due to safety issue	
A Prospective Non-therapeutic Study in Patients Diagnosed With Niemann-Pick Disease Type C [35]	NPC Clinical Severity Score (NPCCSS) [18] 5-Domain NPCCSS [16] NPC-cdb Scale [20]	
Arimoclomol Prospective Study in Patients Diagnosed With Niemann Pick Disease Type C [28]	5-Domain NPCCSS [16]	NPC-cdb Scale [20] NPC Clinical Severity Score (NPCCSS) [18]
A Phase I/II study to evaluate Trappsol Cyclo (hydroxypropyl-ß-cyclodextrin) in patients with Niemann-Pick disease type C (NPC-1) to assess what the drug does to the body, and what the body does to the drug, and the side effects and benefits experienced by patients [36]	NPC Clinical Severity Score (NPCCSS) [18]	NPC Clinical Severity Score (NPCCSS) [18]
Open‐Label Study of Long‐Term Safety and Efficacy of Intravenous Trappsol Cyclo (HPβCD) in Niemann‐Pick Disease Type [37]		NPC Clinical Severity Score (NPCCSS) [18]
Hydroxypropyl Beta Cyclodextrin for Niemann-Pick Type C1 Disease [38]		NPC Clinical Severity Score (NPCCSS) [18]
VTS-270 to Treat Niemann-Pick Type C1 (NPC1) Disease [39]	4-Domain NPCCSS (ambulation, cognition, fine motor, and swallowing)	NPC Clinical Severity Score (NPCCSS) [18]
Study of Lithium Carbonate to Treat Niemann-Pick Type C1 Disease [40]	NPC Clinical Severity Score (NPCCSS) [18]	
Open-label Study of VTS-270 in Participants With Neurologic Manifestations of Niemann-Pick Type C1 [41]		NPC Clinical Severity Score (NPCCSS) [18]
Safety and Efficacy of Miglustat in Chinese NPC Patients [42]		Disease-specific Disability Scale [19]
Adrabetadex for Patients With Nerve Symptoms of Niemann-Pick Type C Disease (NPC) [43]		NPC Clinical Severity Score (NPCCSS) [18]
Longitudinal Study of Cognition With Niemann-Pick Disease, Type C (NPC) [44]	NPC Clinical Severity Score (NPCCSS) [18]	

## Abbreviations

9HPT: 9-Hole Peg Test
NIHR: National Institute for Health Research
NPC: Niemann–Pick disease Type C
NPCCSS: NPC Clinical Severity Score
CNS: Central Nervous System
